# Prognostic Role of TSH Within Euthyroid T2DM Patients with Retinopathy: A 3-Year Cohort Study

**DOI:** 10.3390/diseases13070217

**Published:** 2025-07-12

**Authors:** Nilgun Tan Tabakoglu, Mehmet Celik

**Affiliations:** 1 Hospital Health Practice and Research Center, Faculty of Medicine, Trakya University, Edirne 22030, Turkey; 2 Department of Internal Medicine—Endocrinology and Metabolism, Trakya University, Edirne 22030, Turkey; drmehmetcelik@hotmail.com

**Keywords:** thyrotropin reference range, non-linear association, risk stratification, short-term prognosis, hormonal biomarkers

## Abstract

Background/Objectives: We aimed to determine how baseline TSH levels relate to clinical outcomes over a three-year follow-up in euthyroid patients with T2DR. Methods: This single-center retrospective cohort study included 363 euthyroid T2DR patients who were followed for three years after baseline TSH measurement. Patients were stratified into tertiles based on TSH values belonging to the standard clinical limits (0.35–4.50 mIU/L). Binary and multivariate logistic regression analyses, along with non-linear modeling, were used to evaluate the prognostic impact of TSH and its interaction with age on mortality. The study adhered to the STROBE guidelines. Results: In the first year of follow-up, Group 1 (TSH 0.35–1.24 mIU/L) had significantly higher rates of mortality and combined outcomes compared to Group 2 (TSH 1.24–1.94 mIU/L; *p* = 0.025 and *p* = 0.041, respectively). Group 2 had a lower risk (OR for mortality = 0.349, *p* = 0.004; OR for combined outcome = 0.358, *p* = 0.007). Between TSH and TSH tertiles, a non-linear, inverted U-shaped relationship was observed, with the lowest mortality risk near 2.0 mIU/L. A significant interaction between TSH and age was found for third-year mortality (*p* = 0.016). Conclusions: TSH values showed a non-linear association with outcomes in euthyroid T2DR patients. Group 2 was linked to the lowest risk. Given the significantly higher mortality and combined complications identified within Group 1, closer monitoring and individualized follow-up strategies may be warranted for these patients. Additionally, TSH’s impact on long-term mortality increased with age, supporting its use alongside age for risk stratification in T2DR.

## 1. Introduction

Type 2 diabetes mellitus (T2DM) and thyroid dysfunction are prevalent endocrine diseases in the general population [1].

The pathophysiology of T2DM is characterized by increasing insulin resistance alongside a deterioration of pancreatic β-cell activity over time. Projections from the International Diabetes Federation suggest that the global number of individuals with T2DM may rise to approximately 578 million by 2030 and exceed 700 million by the year 2045 [2,3]. The coexistence of thyroid dysfunction and T2DM is frequently observed across the overall population [4]. A comprehensive analysis compiling data from studies published between 2000 and 2022 estimated that about one in five individuals with T2DM also exhibit some form of thyroid dysfunction [5].

Thyroid hormone signaling has been implicated in both macrovascular and microvascular complications of diabetes. Among the receptors, THRβ is of particular interest due to its expression in retinal and renal tissues [6].

Multiple risk factors have been associated with vascular complications and mortality in T2DM [7]. TSH levels have rarely been evaluated in this context. Some studies have reported associations between thyroid dysfunction and increased morbidity and mortality in diabetic patients, but data on TSH within the euthyroid range remain scarce [5].

Diabetic retinopathy is a frequently encountered microvascular complication of T2DM and stands as the foremost cause of vision loss linked to the disease [8]. This complication occurs as a result of multicomponent pathophysiological processes, including increased capillary permeability, abnormal vascular proliferation, retinal edema, and ischemia due to persistent hyperglycemia. In particular, several investigations have reported that vascular endothelial growth factor, which is expressed secondary to decreased retinal capillary perfusion, contributes significantly to the molecular and cellular events leading to DR [9]. An investigation conducted in 2025 showed that disruptions in tyrosine metabolism might contribute to the underlying mechanisms of DR, and L-tyrosine levels may be used as a predictive biomarker in patients with T2DM [8]. Another endocrine marker that may affect the clinical course of DR is likely to be thyroid hormones, since they determine retinal health in terms of both structure and function. This is because triiodothyronine (T3) is involved in the retinal development and maturation of photoreceptor cells, and through thyroid hormone receptors (especially TRβ1 and TRβ2), it is effective in the differentiation of cone cell subtypes and regulation of opsin gene expression [10]. Therefore, it is thought that alterations in thyroid function may have clinical consequences not only at the systemic level but also at the retinal level. However, the potential effects of thyroid function on disease-related prognosis remain unclear, underscoring the importance of continued investigation in this field. Recent evidence suggests that even within the euthyroid range, variability in TSH levels may be associated with increased risks of adverse outcomes, including cardiovascular mortality and renal dysfunction in patients with type 2 diabetes mellitus [11,12]. Furthermore, the current literature lacks sufficient data regarding the temporal connection between TSH stratification and the probability of macrovascular or other microvascular outcomes, including mortality, in euthyroid patients with T2DR.

This study aimed to determine the safer and riskier TSH ranges regarding mortality or complications during a 3-year follow-up by dividing TSH levels measured at admission into tertiles in euthyroid T2DR patients. We hypothesized that deviations toward either the lower or upper ends of the normal TSH range may correspond to heightened risk, while mid-range TSH levels would be linked to better outcomes.

## 2. Materials and Methods

### 2.1. Study Design and Participants

This study was conducted as a single-center retrospective study in Turkey. The reporting of this study complies with the STROBE (Strengthening the Reporting of Observational Studies in Epidemiology) guidelines for cohort studies. Clinical outcomes were retrospectively evaluated over a three-year follow-up period after the initial TSH measurement.

All deaths and complications were identified retrospectively using the hospital’s electronic health record system, ICD-10 diagnosis codes, laboratory findings, and physician documentation. These endpoints were reviewed and verified by the principal investigator to ensure accuracy. The exact dates of events were extracted from time-stamped records in the hospital system. The follow-up period for each patient began after the first TSH measurement and lasted up to three years, with baseline data collected between 1 January 2019, and 31 December 2019. The study included 363 patients aged 18 years or older with a diagnosis of T2DM for at least 5 years who were followed-up at our hospital’s endocrinology outpatient clinic. Additionally, all patients not receiving insulin therapy were known to be on oral antidiabetic treatment; however, the specific agents or treatment regimens were not recorded.

Participants were enrolled based on the following conditions: aged 18 years or above; having a diagnosis of T2DM for at least five years, based on the American Diabetes Association (ADA) criteria; having a diagnosis of proliferative diabetic retinopathy in at least one eye confirmed by ophthalmologists at our university hospital according to the EURETINA guidelines and being under regular follow-up at the university ophthalmology outpatient clinic; and having TSH levels within the defined euthyroid reference range (0.35–4.5 mU/mL) [13]. Only the first basal TSH values obtained between the study dates were used for analysis; long-term changes in TSH values were not evaluated.

Participants were excluded based on the following conditions: being under 18 years of age; having a TSH level outside the euthyroid reference range defined by the Turkish Society of Endocrinology and Metabolism (TEMD); having one of the microvascular complications of T2DM other than diabetic retinopathy (i.e., diabetic neuropathy or diabetic nephropathy) at the time of enrollment; having any macrovascular complication of T2DM, including cardiovascular diseases; having current or previous levothyroxine replacement therapy; having a history of hypothalamic or pituitary disease; having a comorbid condition unrelated to T2DM but known to affect mortality (e.g., cancer, chronic obstructive pulmonary disease, autoimmune diseases, chronic liver diseases, or neurodegenerative disorders); using medications that may affect thyroid function; pregnancy; or lactation.

The diagnosis of T2DM was confirmed using the 2019 ADA criteria, and DR diagnosis was made based on the EURETINA guidelines [14,15]. Demographic characteristics included age, gender, and smoking status. The medical history was reviewed for diabetes duration (≥5 years), presence and duration of hypertension, and insulin use. Individuals were considered hypertensive if they had a systolic blood pressure of 140 mmHg or higher, a diastolic pressure of at least 90 mmHg, or were receiving treatment with antihypertensive agents [16]. All biochemical analyses were conducted in the central biochemistry laboratory of our institution by certified personnel using validated automated analyzers, in accordance with standard quality control protocols and clinical laboratory accreditation requirements. Blood samples were collected between 8:00 a.m. and 12:00 p.m. after fasting for at least 8 h.

### 2.2. Ethical Approval

The study protocol was reviewed and approved by our university’s Ethics Committee of the Faculty of Medicine (protocol code: TÜTF-GÖBAEK 2024/42; date: 19 February 2024). All procedures were conducted under the ethical framework established by the Declaration of Helsinki and its later modifications.

### 2.3. TSH Groups

Participants were classified into three evenly dispersed subgroups according to their TSH levels. After sorting the TSH values in ascending order, the sample was stratified into tertiles to ensure equal group sizes. This approach was chosen to achieve balanced group sizes for statistical comparison and to facilitate the assessment of potential non-linear associations between TSH levels and outcomes. Additionally, tertile stratification was chosen instead of equal-range grouping to avoid unbalanced group sizes caused by the non-uniform distribution of TSH values and to maintain adequate statistical power across subgroups. Accordingly, the following groups were obtained:

- Individuals with TSH levels ranging 0.35 < TSH < 1.24 mIU/L were classified as Group 1 (n = 120);
- Individuals with TSH levels ranging 1.24 ≤ TSH ≤ 1.94 mIU/L were classified as Group 2 (n = 122);
- Individuals with TSH levels ranging 1.94 < TSH ≤ 4.50 mIU/L were classified as Group 3 (n = 121).

This classification was designed to ensure balanced sample sizes between groups, to evaluate the effect of TSH levels on euthyroid T2DR patients in more detail, and to determine possible risk thresholds.

### 2.4. Statistical Analysis

The Shapiro–Wilk test was employed to ascertain the distribution trend of quantitative variables. The data were summarized using mean and standard deviation values for variables following a normal distribution. In contrast, non-normally distributed variables were summarized using the median, minimum, and maximum values. Frequencies and proportions were used to describe categorical data.

Based on TSH levels, participants were classified into three equal tertile groups. These groups were visualized using box plots, displaying statistical measures such as the median and interquartile range.

To compare quantitative variables among groups, one-way ANOVA, analysis of variance, was applied to variables with a normal distribution, whereas the Kruskal–Wallis test was used when normality assumptions were not met. For categorical comparisons, the chi-square test was applied. To adjust for multiple pairwise comparisons, Bonferroni’s correction was used.

Multivariate logistic regression was employed to examine the association between TSH tertiles and to evaluate the effect of TSH tertiles on one-year mortality and the combined outcome of one-year mortality + microvascular complications. In this study, “microvascular complications” were defined as newly diagnosed or developed cases of diabetic nephropathy or neuropathy during follow-up. “Combined outcome” was defined as the co-occurrence of mortality and microvascular complications in the same patient during the first and third years of follow-up. Independent variables included TSH group (Group 1, Group 2, Group 3), age, sex, smoking history, and insulin use. Findings were reported in terms of odds ratios (ORs) accompanied by 95% confidence intervals (CIs). Model performance in terms of fit was tested via the Hosmer–Lemeshow goodness-of-fit test, and the overall model significance was determined via the Omnibus test.

To examine a potential non-linear (inverted U-shaped) relationship between TSH levels and first-year mortality, a separate logistic regression model was constructed that included both linear (TSH) and quadratic (TSH^2^) terms. Age, gender, insulin use, and smoking status were entered as covariates to evaluate the independent effects of all variables on one-year mortality.

To assess potential interaction effects, an interaction term (TSH × age) was created and tested using binary logistic regression models. Separate models were run for one-year and three-year mortality outcomes. Although no formal subgroup analysis was performed, a descriptive interaction figure (TSH × age) was generated to explore age-dependent mortality patterns. There were no missing data for the variables analyzed. Therefore, no imputation methods were required. This is implicitly ensured by the retrospective design and the use of complete electronic health records.

We adjusted for age, gender, hypertension, insulin use, and smoking, as these are established risk factors for vascular outcomes and mortality in patients with T2DM

Additionally, post hoc power assessments were performed with G*Power (version 3.1.9.4) for both first- and third-year mortality outcomes across TSH tertiles. The calculated effect sizes were w = 0.466 (n = 363) for year 1 and w = 0.369 (n = 294) for year 3. Given α = 0.05 and df = 2, the achieved statistical powers were 100% and 99.9%, respectively, suggesting that the study had enough statistical power to identify relevant differences in mortality outcomes across TSH categories at both follow-up points.

A *p*-value of less than 0.05 was deemed the criterion for statistical significance. All analyses were conducted with the SPSS 20.0 software (IBM SPSS Statistics for Windows, Version 20.0, Armonk, NY, USA). The predicted probability curve of mortality risk across TSH levels was visualized using Python 3.10 with the statsmodels and matplotlib libraries.

## 3. Results

The participants had an average age of 62.9 ± 9.3 years, as indicated in Table 1, with females representing 39.9% of the sample.

**Figure 1:**
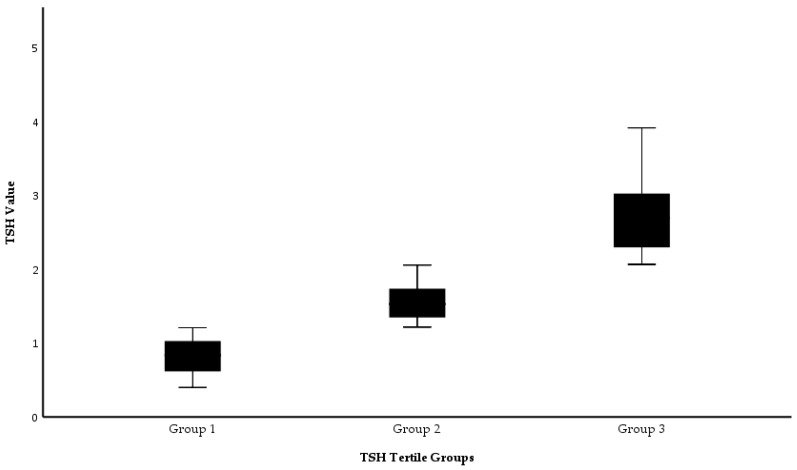
Distribution of TSH levels across tertile groups. Box plots are used to visually represent the median, interquartile range, and full range. A statistically significant difference was observed between the groups (*p* < 0.001, Kruskal–Wallis test); TSH: thyroid-stimulating hormone; mIU/L: milli-international units per liter.

The distribution of TSH values across the tertile groups is presented in Figure 1. A statistically significant difference in TSH levels was observed between the groups (*p* < 0.001, Kruskal–Wallis test).

An analysis of patient-related demographic and medical features among the patient groups according to TSH tertiles is presented in Table 2. The statistical analysis revealed a significant distinction in free thyroxine (FT4) levels among the groups (*p* = 0.023); this difference was attributed to higher FT4 levels in the first tertile group compared to the third tertile group (*p* = 0.018, Bonferroni’s correction). No other significant differences were found among the tertile groups (*p* > 0.05).

A comparison of the mortality and complication values of the patients at one and three years is presented in Table 3. At one year, a considerable discrepancy was noted in mortality between the groups (*p* = 0.025), primarily driven by a higher mortality incidence observed in the lowest TSH tertile compared to the second. Likewise, combined mortality and microvascular complications differed significantly between groups (*p* = 0.041), again attributable to elevated event rates in the first tertile relative to the second. No statistically meaningful differences were identified among tertile groups for the remaining parameters (*p* > 0.05).

The effects of TSH groups on one-year mortality and one-year mortality + microvascular complications were evaluated using multivariate logistic regression analysis, as presented in Table 4. According to the results, TSH groups had a statistically significant impact on both one-year mortality and one-year mortality + microvascular complications (*p* = 0.015 and *p* = 0.026, respectively).

Patients in TSH Group 2 had a lower mortality risk than those in Group 1 (B = −1.053, *p* = 0.004, OR = 0.349, 95% CI: 0.195–0.778). This group also demonstrated a protective effect against the combined outcome of mortality + microvascular complications (B = −1.027, *p* = 0.007, OR = 0.358, 95% CI: 0.169–0.759). In contrast, no significant association was found between TSH Group 3 (1.94–4.50 mIU/L) and either mortality or the development of microvascular complications (mortality: B = −0.319, *p* = 0.323; mortality + microvascular complications: B = 0.251, *p* = 0.453).

Age was identified as the strongest independent predictor for both mortality (B = 0.054, *p* = 0.001, OR = 1.056, 95% CI: 1.020–1.089) and mortality + microvascular complications (B = 0.046, *p* = 0.005, OR = 1.047, 95% CI: 1.014–1.081). Gender, smoking status, and insulin use were not found to be significantly associated with mortality or microvascular complications (*p* > 0.05). The overall significance of the models was confirmed by the Omnibus test (*p* = 0.001 and *p* = 0.005, respectively), and the goodness-of-fit was evaluated as acceptable based on the Hosmer–Lemeshow test (*p* = 0.543 and *p* = 0.406, respectively). In conclusion, Group 2 patients with intermediate TSH levels had a lower risk of 1-year mortality and microvascular complications, while age was the most critical risk factor. A summary of the multivariate regression model results is presented in Table 4.

**Figure 2:**
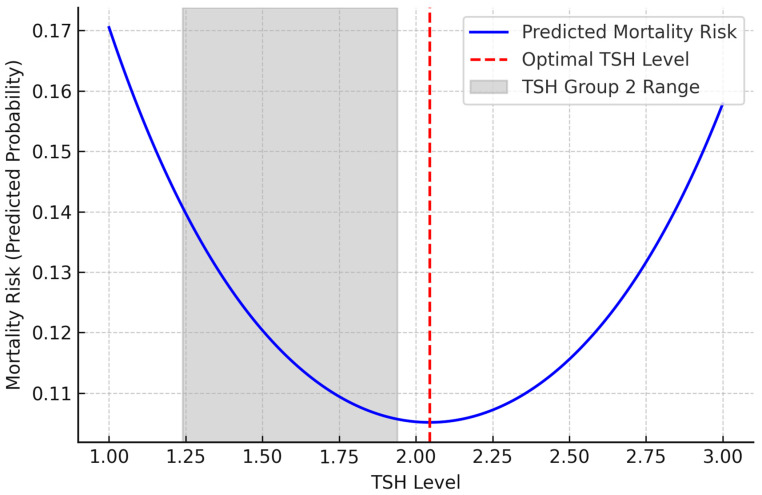
Inverse U-shaped correlation observed with serum TSH levels and one-year mortality risk, X-axis: TSH levels; Y-axis: mortality risk (predicted probability); blue line: predicted mortality risk across TSH levels; red dashed line: optimal TSH level where mortality risk is lowest, gray-shaded area: TSH Group 2 (1.24–1.94 mIU/L).

In the non-linear logistic regression analysis, the linear coefficient of TSH was negative (B = −2.094, *p* = 0.002), while the quadratic term (TSH^2^) was positive (B = 0.512, *p* = 0.002). These findings support a non-linear, inverted U-shaped relationship between TSH levels and one-year mortality (Table 5). This relationship is visualized in Figure 2. Accordingly, the mortality risk was higher at lower TSH levels, decreased at intermediate levels, and increased again at higher levels. Furthermore, as shown in Figure 2, the regression curve reveals a non-linear, inverted U-shaped relationship between TSH levels and 1-year mortality risk, with the lowest risk observed around 2.0 mU/L, within the upper range of Group 2. This relationship is visualized in Figure 2.

**Figure 3:**
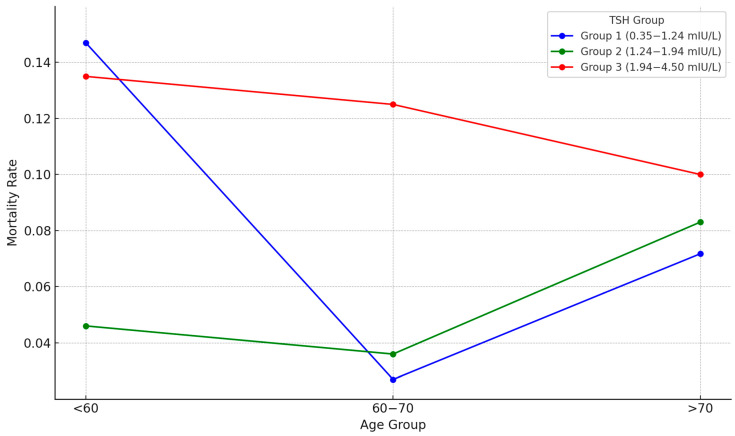
Age-stratified 3–year mortality rates across TSH tertile groups. Intermediate TSH levels (Group 2: 1.24–1.94 mIU/L) were consistently associated with lower mortality across all age strata.

Binary logistic regression was employed to assess the interaction between TSH and age, revealing no significant association in regression models incorporating this variable for death in the first year (*p* = 0.976). However, the interaction was significant for third-year mortality (*p* = 0.016), indicating that age modifies the effect of TSH on long-term outcomes. To visually illustrate the interaction between age and TSH groups on third-year mortality, a descriptive line plot was constructed (Figure 3).

## 4. Discussion

The primary objective of this research was to explore how varying TSH levels may influence the risk of mortality, as well as the development of microvascular and macrovascular complications, during a three-year observational period in euthyroid patients with DR and a minimum five-year history of T2DM. Participants were categorized according to their serum TSH concentrations, and outcomes such as mortality and complications were examined at two distinct time points—specifically, at the end of the first and third years of follow-up. The findings from these intervals were compared to determine how clinical trajectories differed in relation to baseline TSH levels.

Our findings revealed significant differences in first-year mortality and combined mortality + microvascular complications among the TSH tertile groups; notably, Group 1, which included patients with the lowest TSH levels, demonstrated significantly higher rates (Table 2, *p* = 0.025 and *p* = 0.041, respectively). These results suggest that even within the euthyroid range, TSH may be a key factor influencing the disease course in patients diagnosed with T2DR. Furthermore, the lowest mortality rates were observed in Group 2, with TSH levels ranging from 1.24 to 1.94 mIU/L (inclusive). This may indicate that this range represents a safer threshold regarding metabolic stability and vascular integrity. In contrast, although Group 3 (TSH 1.94–4.50 mIU/L) exhibited higher mortality rates compared to Group 2, no meaningful statistical difference was observed. These results demonstrate a non-linear (inverted U-shaped) relationship between TSH levels and clinical outcomes, highlighting the potential prognostic significance of maintaining TSH within an optimal range, even in euthyroid patients with T2DR.

In addition, patients in the lowest TSH tertile (Group 1) were found to have significantly higher FT4 levels compared to those in the highest TSH tertile (Group 3), (Table 2; *p* = 0.023). This hormonal profile may reflect a subclinical thyrotoxic state, as lower TSH accompanied by relatively elevated FT4 has been linked to vascular vulnerability. In a study evaluating patients with subclinical thyrotoxicosis, impaired endothelial function was demonstrated through reduced circulating endothelial progenitor cells (cEPCs) and lower flow-mediated dilation, both of which are essential for vascular repair [17]. Additionally, in vitro experiments have indicated that T3 may trigger early apoptotic pathways and downregulate endothelial nitric oxide synthase in vascular tissues, potentially compromising endothelial integrity. These mechanisms may help explain the increased early mortality and microvascular complications observed in Group 1, despite the absence of overt thyrotoxicosis.

The actions of thyroid hormones are mediated by nuclear receptors (THRα and THRβ) that are present in multiple tissues such as the cardiovascular system, retina, liver, and pancreas [18,19,20]. By influencing metabolic processes and vascular function, thyroid hormones may potentially contribute to the development and progression of diabetes-related complications [21]. However, the complex mechanisms underlying these interactions remain a mystery.

THRβ is predominantly present in the retina under normal conditions and shows high expression in cone cells. This information is relevant to understanding the mechanisms associated with DR. Thyroid hormone signaling via THRβ is believed to have a crucial function in initiating and progressing changes related to retinal vascular architecture [22]. These observations imply that thyroid hormones could impact both systemic metabolic processes and diabetes-associated tissue injury, a conclusion that aligns with the results observed in our study. Nonetheless, it is important to highlight that the development and progression of diabetic retinopathy are predominantly influenced by sustained hyperglycemia and inherent genetic factors. Therefore, the prognostic association identified between TSH fluctuations and clinical outcomes in our study should be interpreted as secondary and relatively limited compared to these well-established contributors [23,24].

In our investigation of euthyroid individuals with T2DR, the average age was calculated as 62.9 ± 9.3 years, and females constituted 39.9% of the study population (Table 1). According to previously published data, the percentage of female patients with T2DR typically falls within the range of 38.7% to 45.1% [25,26]. This similarity shows that the gender distribution of our patient population overlaps with the sample groups in the literature.

In an investigation of 1024 individuals diagnosed with type 2 diabetes, hypertension and hyperlipidemia were identified as the most prevalent comorbid conditions, affecting 84.9% of the participants [27]. In our study, 70.2% of the patients used insulin, and 94.2% had hypertension; these rates were found to be compatible with the literature. Earlier research has indicated that hypertension frequently coexists with type 2 diabetes mellitus and serves as a major contributor to the development of vascular complications [28].

Moreover, 51.5% of the individuals in our study had a smoking history equal to or exceeding 20 pack-years. This observation aligns with the existing literature suggesting that smoking substantially contributes to the advancement of both microvascular and macrovascular complications in diabetic patients [29]. These data from our study indicate that comorbidities serve as a key contributor during T2DM, and hypertension and smoking are prominent risk factors.

T2DM is defined as a chronic disturbance in glucose metabolism stemming from impaired insulin secretion, peripheral insulin resistance, or a combination of these pathophysiological processes [30]. The resulting persistent hyperglycemia increases oxidative stress by increasing the production of reactive oxygen species (ROS) through pathways such as AGE formation, mitochondrial dysfunction, and polyol shunting. This imbalance impairs endothelial function and promotes inflammation, contributing to both microvascular and macrovascular complications in diabetic patients [31].

T2DR is a microvascular complication in which chronic inflammation and oxidative stress play key roles. Hyperglycemia promotes AGE formation and nuclear factor kappa B activation, while depleting NADPH via the polyol pathway, leading to excess ROS and retinal damage. NOX enzymes further increase ROS production, contributing to vascular leakage and neovascularization [32]. Additionally, lipid peroxidation, elevated fatty acid binding protein 4, and impaired antioxidant responses exacerbate oxidative stress in T2DR [33].

The progression of T2DR has been strongly correlated with elevated inflammatory activity. Individuals diagnosed with T2DR exhibit significantly increased inflammatory mediator levels, such as TNF-α, IL-1β, IL-6, IL-8, and IL-17A. In a hyperglycemic environment, the expression of these cytokines has been shown to trigger endothelial dysfunction, increased vascular permeability, and neovascularization [34]. Additionally, stimulation of the NLRP3 inflammasome signaling cascade and overexpression of thioredoxin-interacting protein has been reported to accelerate pericyte apoptosis, disrupt retinal vascular integrity, and contribute to the progression of T2DR [35].

TSH exerts its effect by binding to THRs in the thyroid gland [36]. Recently, studies have shown that both altered TSH levels and subclinical hypothyroidism have been linked to T2DR in diabetic patients [37,38]. A cohort study of 1938 participants from the Tehran Thyroid Study group showed a complex relationship between changes in thyroid hormones and T2DM [39].

The study demonstrated that both mortality and microvascular complications were linked to short-term outcomes in patients diagnosed with T2DR (Table 3). Group 1, characterized by the lowest TSH concentrations, exhibited a significantly increased mortality rate (Table 3, *p* = 0.025). Although the overall distribution regarding micro–macrovascular complications was similar, a meaningful difference across groups was observed concerning microvascular complications + mortality rate (Table 3, *p* = 0.041). This observation aligns with evidence reported in a 2022 investigation showing that TSH levels, despite being within normal ranges, were significantly correlated with mortality due to all causes, as well as cardiovascular events in euthyroid patients [11]. Another study in euthyroid T2DM patients showed that a notable correlation was identified between TSH concentrations and reduced eGFR values [12]. This finding of our study suggests that in euthyroid patients with T2DR, reduced TSH levels may correlate with elevated rates of death and microvascular poor effects. This may be elucidated by the interaction of thyroid hormones with inflammatory processes, oxidative stress, and vascular dysfunction. In the existing literature, low TSH levels have been shown to increase endothelial dysfunction by impairing anti-inflammatory mechanisms and exacerbating the already high levels of oxidative stress in diabetic patients [40]. As a result, accelerated inflammation and vascular damage within the first year may have given rise to a meaningful surge in both mortality and concomitant microvascular complications in the early period, and our result is consistent with the existing literature [40].

However, in the long term, this difference may have stabilized over time due to factors such as changes in medical treatment approaches, metabolic control of patients, and survival bias. In the literature, in a large-scale study of 3515 coronary artery disease patients with TSH concentrations within reference limits stratified into three subgroups based on their TSH concentrations, thyroid function was shown to be closely interconnected with cardiac and vascular mechanisms and inflammatory processes; it was reported that major cardiovascular events and heart failure developed more frequently, especially in patients with low and high TSH levels, so TSH can be considered as an independent prognostic marker [41]. These results are in line with what we found, which show the association between TSH levels and vascular dysfunction and inflammatory response in diabetic individuals. They also support the idea that TSH may be a marker not only of thyroid function but also of cardiometabolic risk.

Multivariable logistic regression analysis of the groups according to TSH levels showed that Group 2 patients had a significantly lower risk of both 1-year mortality and mortality + microvascular complications compared to the reference Group 1 (B = −1.053, *p* = 0.004 and B = −1.027, *p* = 0.007; Table 4). In contrast, no significant difference was observed for these two clinical outcomes in Group 3. These findings suggest that intermediate TSH levels (1.24–1.94 mU/mL) may represent a clinically safer range. Furthermore, TSH has demonstrated a statistically significant correlation with age, mortality, and the emergence of mortality alongside microvascular problems (B = 0.054, *p* = 0.001; B = 0.046, *p* = 0.005), indicating that age constitutes an independent risk factor for clinical prognosis (Table 4). This finding aligns with a 2022 study examining the correlation between TSH levels and microvascular complications in 248 euthyroid T2DM patients [42]. This study reported that thyroid hormone levels and age were determinants in the development of microvascular complications.

In our study, in the multivariate logistic regression analysis evaluating the clinical variables that may be effective in predicting mortality at 1 year, the linear coefficient of TSH was negative (B = −2.094, *p* = 0.002), and the quadratic (TSH^2^) coefficient was positive (B = 0.512, *p* = 0.002, Table 5). These findings suggest a non-linear, inverted U-shaped relationship between TSH levels and 1-year mortality (Figure 2). According to this model, the lowest mortality risk was observed at a TSH level of approximately 2.0 mU/L. However, when tertile groups were formed according to TSH levels in our study, Group 2, which covers the TSH range of 1.24–1.94 mU/L, was associated with a significantly lower mortality risk compared to both Group 1 and Group 3. Interestingly, the minimum point on the curve, TSH 2.0, was located in Group 3, but since the majority of individuals in this group had TSH values above 2.0, mortality risk increased again across the group.

This shows that the regression model can show the effect of TSH as a continuous variable with precision, but average trends are prominent in group-based analyses. Therefore, the identification of Group 2 as the most advantageous group in terms of mortality may be because individuals in this range are both close to the optimum value in the model and clustered in a narrower safe range.

In other words, the risk of mortality increases in Group 1 patients with very low TSH levels, decreases to the lowest level in Group 2 patients with intermediate levels and increases again in Group 3 patients with higher TSH levels (Figure 2). Similarly, a 2023 study involving 422 euthyroid T2DM patients found an inverted U-shaped non-linear relationship between thyroid hormone sensitivity and DR risk [25]. These results suggest that Group 2 TSH levels may have a protective effect on short-term survival.

This non-linearity suggests that TSH may not only be a parameter reflecting thyroid function but also a prognostic marker associated with systemic inflammation, vascular stress, and metabolic balance in individuals with diabetes. Furthermore, the significant positive association between age and mortality (Table 5; B = 0.054, *p* = 0.001) supports the impact of age-related vascular dysfunction on prognosis in patients with T2DR. This finding suggests that the increasing systemic risk associated with aging may be a critical determinant of survival in individuals with T2DR. The other variables of gender, insulin use, and smoking history were not found to be significant in our analysis; this supports that TSH level may be an independent risk indicator (Table 5).

This study concludes that in euthyroid patients with T2DR, TSH levels within the normal reference range exhibit a non-linear relationship with the risk of mortality and microvascular complications. Low TSH levels were specifically linked to a heightened risk of mortality and microvascular complications during the initial year of follow-up. Additionally, the significant interaction between TSH and age observed in the third-year analysis suggests that the prognostic impact of TSH levels on mortality becomes more pronounced with advancing age. This relationship was not evident in the first-year outcomes, possibly due to the shorter observation time or survival bias. Visual trends in mortality across age groups and TSH tertiles further support the hypothesis that the prognostic impact of TSH is modified by age (Figure 3). These outcomes suggest that incorporating patient age into risk assessment is essential when evaluating the impact of TSH levels on mortality, especially over extended follow-up periods. These findings suggest that TSH is not only a biochemical parameter reflecting thyroid function but also a potential marker that may influence clinical prognosis in euthyroid patients with T2DR. Clinically, our results indicate that euthyroid T2DR patients in Group 1 (TSH 0.35–1.24 mIU/L) may carry an elevated risk profile. These patients could benefit from closer follow-up, including regular assessments of glycemic status, renal function, and early microvascular changes. While no direct treatment is indicated for low–normal TSH, identifying this group may support earlier risk mitigation. Further studies are needed to confirm these thresholds and their utility in guiding patient management.

However, this investigation is subject to certain limitations. The single-center and retrospective design restricts the generalizability of the findings. One of the main limitations of this study is that although all patients were diagnosed with proliferative diabetic retinopathy based on ophthalmologic evaluation, detailed parameters such as the extent of retinal involvement, visual acuity, and treatment history (e.g., photocoagulation or anti-VEGF therapy) were not included in the analysis. However, these data were deliberately excluded, as the primary aim of the study was not to assess ophthalmologic outcomes but rather to evaluate the relationship between TSH levels and mortality as well as systemic micro- and macrovascular complications in patients with type 2 diabetes and diabetic retinopathy. Finally, despite careful exclusion criteria and baseline assessments, we recognize that unmeasured factors such as subclinical inflammation, nutritional deficiencies, or chronic stress could have influenced thyroid parameters. This residual confounding factor remains an inherent limitation of retrospective observational studies. Although our results are consistent with previous research and supported by robust statistical analyses, they should be interpreted with caution. Future multicenter studies involving larger and more diverse populations are necessary to validate these findings. These studies could enhance the formulation of personalized and risk-oriented follow-up strategies for patients with diabetic retinopathy by facilitating the incorporation of TSH levels into clinical decision-making frameworks.

## 5. Conclusions

In our study, in euthyroid T2DR patients, the rates of death and death combined with microvascular complications in the first year of follow-up were significantly higher in Group 1. In contrast, this risk was lowest in Group 2. A non-linear, inverted U-shaped relationship was observed between TSH and mortality in the first year. These findings suggest that TSH levels may predict clinical outcomes in euthyroid T2DR patients, even when within the normal reference range. They also indicate that individuals in Group 1 should be monitored closely, while the Group 2 range may represent a potential clinical target. Furthermore, the significant interaction observed between TSH and age in the third-year analysis highlights that the prognostic impact of TSH increases with advancing age, emphasizing the need for age-specific interpretation of TSH-related risk in long-term follow-up. Regular assessment of TSH levels in patients with type 2 diabetic retinopathy—even when within the normal range—may yield important prognostic insights. Categorizing individuals based on their TSH profiles could enhance personalized risk stratification and guide tailored clinical management strategies. To confirm these findings, the pathophysiological mechanisms underlying the association between TSH levels in the subclinical range and diabetic complications and mortality should be clarified, and whether targeted interventions improve patient outcomes should be determined. Future multicenter prospective studies are required. These observations should, therefore, be interpreted cautiously and not be considered as definitive recommendations without further prospective evidence.

## Figures and Tables

**Table 1:** Clinical and demographic attributes of participants.

	N = 363
Age, years	62.9 ± 9.3
Gender, female	145 (39.9)
Insulin therapy, yes	255 (70.2)
HT, yes	342 (94.2)
Smoking ≥ 20 pack-years	187 (51.5)

Mean ± standard deviation or n (%); HT, hypertension.

**Table 2:** Comparison of clinical and demographic features among TSH tertile subgroups.

	Group 1 (n = 120) TSH: 0.35–1.24 mIU/L	Group 2 (n = 122) TSH: 1.24–1.94 mIU/L	Group 3 (n = 121) TSH: 1.94–4.5 mIU/L	*p*
Age, years	63.1 ± 9.5	64.2 ± 8.6	61.7 ± 9.7	0.119 ^a^
Fasting plasma glucose, mg/dL	169 (68–478)	158.5 (48–386)	175 (50–453)	0.570 ^b^
Urea, mg/dL	45 (12–159)	48 (12–253)	41 (16–278)	0.234 ^b^
Creatinine, mg/dL	1 (0.4–7.8)	1 (0.5–6.8)	1 (0.5–55)	0.926 ^b^
eGFR, mL/min/1.73 m^2^	67.6 (4.9–126.6)	69.5 (5.1–169.2)	67.9 (3.1–151.6)	0.704 ^b^
CRP, mg/dL	0.4 (0.1–67.5)	0.5 (0.1–72.8)	0.5 (0.2–13.4)	0.265 ^b^
ALT, IU/L	17 (4–124)	15 (4–252)	17 (3–91)	0.120 ^b^
AST, IU/L	18 (8–274)	18 (9–124)	19 (7–108)	0.442 ^b^
Albumin, g/L	40 (4.3–49)	41 (2.8–48)	40 (4–48)	0.880 ^b^
Uric acid, mg/dL	5.7 (1.5–10.8)	5.5 (0.6–35)	5.4 (2.4–12.8)	0.784 ^b^
Total bilirubin, mg/dL	0.4 (0.1–2.4)	0.4 (0.1–9)	0.5 (0.1–4)	0.938 ^b^
Total cholesterol, mg/dL	177 (69–353)	184.5 (86–361)	173 (82–356)	0.150 ^b^
Triglycerides, mg/dL	125 (35–645)	132 (38–619)	138 (39–902)	0.537 ^b^
LDL cholesterol, mg/dL	110 (22–229)	119.5 (39–247)	110 (30–291)	0.065 ^b^
HDL cholesterol, mg/dL	42 (19–86)	42 (24–94)	39 (16–85)	0.237 ^b^
Free T4, ng/dL	1.1 (0.5–3.9)	1.1 (0.6–2.16)	1.0 (0.5–2.64)	0.023 **^b^**
T3, ng/mL	2.9 (0.5–4)	2.9 (1–4.1)	2.9 (0.9–5.7)	0.911 ^b^
WBC, 10^3^/µL	8.1 (3.5–19.8)	7.5 (3.9–16.6)	8.3 (4–20.1)	0.119 ^b^
Hemoglobin, g/dL	12.3 (6.7–16.8)	12.4 (1–16.4)	12.3 (7.5–16.7)	0.973 ^b^
HbA_1_c, %	8.3 (5.4–14.2)	8.2 (5.3–15.1)	8.6 (5.5–15.2)	0.260 ^b^
Platelets, 10^3^/µL	236.5 (68–586)	232.5 (85–459)	231 (22–491)	0.546 ^b^
Lymphocytes, 10^3^/µL	1.8 (0.5–5.1)	1.8 (0.5–4.2)	2.1 (0.6–5.9)	0.094 ^b^
Neutrophils, 10^3^/µL	4.9 (1.1–16.7)	4.6 (2–12.7)	5 (0–16.5)	0.373 ^b^
Monocytes, 10^3^/µL	0.6 (0.2–1.6)	0.6 (0.3–1)	0.6 (0–1.5)	0.120 ^b^
Gender, female, yes n (%)	41 (34.2)	52 (42.6)	52 (43.0)	0.287 ^c^
Insulin therapy, n (%), yes	84 (70.0)	86 (70.5)	85 (70.2)	0.997 ^c^
Hypertension, n (%), yes	112 (93.3)	118 (96.7)	112 (92.6)	0.336 ^c^
Smoking ≥20 pack-years, n (%), yes	63 (52.5)	63 (51.6)	61 (50.4)	0.948 ^c^

Mean ± Standard Deviation, Median (minimum–maximum), n (%); ALT, alanine aminotransferase; AST, aspartate aminotransferase; CRP, C-reactive protein; DM, diabetes mellitus; eGFR, estimated glomerular filtration rate; FT4, free thyroxine; HbA_1_c, glycated hemoglobin; HDL-C, high-density lipoprotein cholesterol; LDL-C, low-density lipoprotein cholesterol; PLT, platelet count; SII, systemic immune-inflammation index; T3, total triiodothyronine; TSH, thyroid-stimulating hormone; WBC, white blood cell count. ^a^ One-way ANOVA test; ^b^ Kruskal–Wallis test; ^c^ Pearson’s Chi-square test.

**Table 3:** Comparison of patients’ complication rates and mortality at one- and three-year follow-up, stratified by TSH tertiles.

Variable	Group 1 (n = 120) TSH: 0.35–1.24 mIU/L	Group 2 (n = 122) TSH: 1.24–1.94 mIU/L	Group 3 (n = 121) TSH: 1.94–4.5 mIU/L	*p* ^a^
Death at year 1, yes	30 (25.0)	14 (11.5)	23 (19.0)	0.025
Death at year 3, yes	7 (7.9)	6 (5.6)	11 (11.3)	0.317
Microvascular complication, year 1, yes	92 (76.7)	88 (72.1)	88 (72.7)	0.684
Microvascular complication, year 3, yes	69 (57.5)	83 (68)	77 (63.6)	0.234
Macrovascular complication, year 1, yes	55 (45.8)	63 (51.6)	56 (46.3)	0.602
Macrovascular complication, year 3, yes	37 (30.8)	43 (35.2)	41 (33.9)	0.758
Micro + macro complications, year 1, yes	50 (41.7)	52 (42.6)	46 (38.0)	0.743
Micro + macro complications, year 3, yes	33 (27.5)	36 (29.5)	38 (31.4)	0.802
Death + microvascular complication, year 1, yes	26 (21.7)	12 (9.8)	21 (17.4)	0.041
Death + microvascular complication, year 3, yes	7 (5.8)	6 (4.9)	9 (7.4)	0.707
Death + macrovascular complication, year 1, yes	21 (17.5)	10 (8.2)	15 (12.4)	0.093
Death + macrovascular complication, year 3, yes	5 (4.2)	5 (4.1)	10 (8.3)	0.266
Death + micro + macro complications, year 1, yes	19 (15.8)	8 (6.6)	13 (10.7)	0.070
Death + micro + macro complications, year 3, yes	5 (4.2)	5 (4.1)	9 (7.4)	0.411

n (%), ^a^ Pearson’s chi-square test.

**Table 4:** Effects of TSH tertiles and other covariates on first-year mortality and combined mortality + microvascular complications (multivariate logistic regression).

Variable	Mortality Beta (B)	Mortality *p*-Value (Sig.)	Mortality + Microvascular Complications Beta (B)	Mortality + Microvascular Complications *p*-Value (Sig.)
TSH percentile group (overall effect)	-	0.015	-	0.026
TSH group 2	−1.053	0.004	−1.027	0.007
TSH group 3	−0.319	0.323	0.251	0.453
Gender, female	0.358	0.294	0.454	0.198
Age, years	0.054	0.001	0.046	0.005
Smoking ≥ 20 pack-years, yes	−0.421	0.218	−0.362	0.306
Insulin therapy, yes	−0.201	0.517	0.232	0.477
Constant	−4.467	0.000	−4.414	0.000

B, unstandardized beta; mIU/L, milli-international units per liter.

**Table 5:** Independent effects of TSH, TSH^2^, and clinical covariates on first-year mortality (non-linear logistic regression model).

Variable	Beta (B)	*p*-Value (Sig.)
TSH^2^	0.512	0.002
TSH	−2.094	0.002
Age, years	0.054	0.001
Insulin therapy, yes	0.189	0.541
Gender, female	0.269	0.428
Smoking ≥ 20 pack-years, yes	−0.321	0.345

B, unstandardized regression coefficient; DM, diabetes mellitus. TSH^2^: quadratic term of TSH.

## Data Availability

Our study data contain personal information of patients and, therefore, are not available for sharing due to the ‘Personal Data Protection Law’ and ethical reasons.
